# Pre-immature dendritic cells (PIDC) pulsed with HPV16 E6 or E7 peptide are capable of eliciting specific immune response in patients with advanced cervical cancer

**DOI:** 10.1186/s12967-014-0353-4

**Published:** 2014-12-16

**Authors:** Osama E Rahma, Vincent E Herrin, Rami A Ibrahim, Anton Toubaji, Sarah Bernstein, Omar Dakheel, Seth M Steinberg, Rasha Abu Eid, Mikayel Mkrtichyan, Jay A Berzofsky, Samir N Khleif

**Affiliations:** Cancer Vaccine Branch, CCR, NCI, 10 Center Drive, Bethesda, MD 20892 USA; Walter Reed National Military Medical Center, 8901 Wisconsin Ave, Bethesda, MD 20814 USA; Biostatistics and Data Management Section, CCR, NCI, 9609 Medical Center Drive, Rockville, MD 20850 USA; University of Virginia, Charlottesville, VA 22908 USA; Georgia Regents University Cancer Center, 1411 Laney Walker Blvd, Augusta, GA 30912 USA

**Keywords:** Dendritic cells, HPV16, E6, E7, Vaccine, Cervical cancer

## Abstract

**Background:**

The protein products of the early genes E6 and E7 in high-risk HPV types 16 and 18 have been implicated in the oncogenic capability of these viruses. Therefore, these peptides represent attractive vaccine therapy targets.

**Methods:**

Thirty-two patients with advanced cervical cancer (HPV16 or 18 positive) were treated with HPV16 E6 (18–26) (Arm A) or HPV16 E7 (12–20) peptide (Arm B) pulsed on PBMCs in order to illicit immune response against the relevant peptide on both arms. These PBMCs were cultured for a short time (48 hours only) and in the presence of GM- CSF, accordingly, they were identified as “Pre-Immature Dentritic Cells”.

**Results:**

51Cr release assay and ELISPOT demonstrated evidence of specific immune response against the relevant peptide in 10/16 (63%) evaluable patients in arm A and 7/12 (58%) in arm B. HPV16 E6 was found to be homologous to HPV18 E6 in both *vivo* and *vitro*. The median overall survival (OS) and progression free survival (PFS) for the full cohort was 10.0 and 3.5 months, respectively. There were no RECIST responses in any patient. The majority of toxicities were grade I and II.

**Conclusions:**

We demonstrated the feasibility and ability of Pre-Immature Dentritic Cells pulsed with HPV16 E6 (18–26) or HPV16 E7 (12–20) to induce a specific immune response against the relevant peptide despite the advanced disease of the cervical cancer patients treated on this trial. We believe that this observation deserves further investigations.

**Electronic supplementary material:**

The online version of this article (doi:10.1186/s12967-014-0353-4) contains supplementary material, which is available to authorized users.

## Background

Cervical cancer is the third most common malignancy in women and the fourth leading cause of female cancer-related death worldwide [1]. More than 500,000 new cases of invasive cervical cancer are diagnosed throughout the world yearly, accounting for over 275,000 deaths [1]. High-risk human papillomavirus (HPV) has been implicated as an etiologic factor in almost all cervical cancers [2]. Current therapeutic strategies for advanced cervical cancer have proven ineffective [3,4]. The recent development and FDA approval of two vaccines for HPV infection have represented a major advance in cervical cancer prevention [5-8]. However, the effect of prophylactic vaccines on women already infected with HPV remains to be determined, and the impact of prophylactic vaccines on the elimination of cervical cancer is not expected to be apparent for decades [9]. Accordingly, better therapeutic modalities for advanced cervical cancer are needed.

HPV16 and HPV18 are the two most carcinogenic HPV types amongst the high-risk HPV, and they are responsible for about 70% of invasive cervical cancer cases [10,11]. HPV-E6 and E7 are the main oncogenic protein products of high risk viruses and their continuous expression is necessary for the initiation and maintenance of malignant transformation [12-14]. Therefore, these antigens represent attractive targets for therapeutic cancer vaccines.

The methods of peptide vaccine administration have varied. One way of delivery is by pulsing the peptide on dendritic cells (DCs), which are the most potent professional antigen-presenting cells [15-17]. Dendritic cells could be prepared as mature or immature cells. Immature DCs are Peripheral Blood Mononuclear Cells (PBMCs) cultured for 5–7 days in GM-CSF and IL-4. On the other hand, mature DCs are usually cultured with the addition of pro-inflammatory cytokines for an additional 24–48 hours [18,19]. Immature DCs can effectively capture antigens but, as opposed to mature DCs, they lack the full T cell co-stimulatory activity [20]. Therefore, most trials that utilized DCs used the mature type [17,21,22]. It is not clear whether maturation of DCs is necessary to generate the needed immune response in cancer vaccines. Furthernore, the preparation of DCs is time consuming, expensive, and requires specialized facilities. Accordingly, here, we tested the feasibility of using PBMCs in a state prior to being immature DCs; “pre immature DCs”. PBMCs were cultured for a shorter time (48 hours only) in the presence of GM- CSF only and used to administer peptides E6 (18–26), or E7 (12–20), as cancer vaccines, to patients with advanced cervical cancers. We found that this method of vaccination is well tolerated and capable of eliciting a specific immune response against the relevant peptide.

## Methods

### Patient selection

Patients were assigned to two arms. Arm A received HPV16 E6 (18–26) peptide, while patients on arm B received HPV16 E7 (12–20) peptide pulsed on pre-immature dendritic cells (PIDCs). Patients had histologically proven advanced cervical carcinoma, recurrent or persistent disease despite prior treatment, and no evidence of brain metastases. Patients on both arms had HLA.A2.1 subtype. Patients who received the E6 peptide harbored either HPV16 or 18 in their tumors, whereas patients who received the E7 peptide harbored HPV16 (Tables 1 and 2). These selection criteria were based on our pre-clinical studies which showed that HPV16 E6 (18–26) and HPV18 E6 (13–21) peptides are homologous. The sequence of both peptides is identical except for amino acid number 21 where glutamine in HPV16 E6 (18–26) is replaced by aspartic acid in HPV 18 E6 (13–21). All enrolled patients met the protocol eligibility criteria, including ECOG performance status of 0–1 and life expectancy of more than 3 months. Main exclusion criteria included history of autoimmune disease, and history of other malignancies except basal cell carcinoma of the skin. Both the National Cancer Institute (NCI) and National Naval Medical Center (NNMC) Institutional Review Boards (IRBs) approved the protocol and the patients’ consent was obtained prior to enrollment.

**Table 1:** **HPV16 E6 patient profile, clinical and immunological outcome**

**Pt**	**Age**	**Path**	**HPV type**	**Disease extension**	***# Vac***	***Off-Study response/reason***	***PFS (ms)***	***OS (ms)***	***Cr-51***	***ELISPOT***	***IR***
1A	39	ACA	18	Mets	8	PD	8.0	20.1	**-**	**+**	**+**
2A	46	SCC	16	Mets	1	SD/PPS	0.7+	1.4	ND	ND	ND
3A	41	SCC	18	Mets	6	PD	5.9	18.6	-	-	-
4A	37	SCC	18	Mets	4	PD	3.6	9.7	**-**	**+**	**+**
5A	47	SCC	18	Mets	2	SD/PPS	2.8+	4.9	-	-	-
6A	36	ASCC	16	Mets	3	PD	1.6	8.7	**+**	**NA**	**+**
7A	40	ASCC	18	Mets	4	PD	4.6	6.0	-	-	-
8A	48	ACA	18	Mets	4	PD	3.4	10.0	**-**	**NA**	**-**
9A	40	SCC	18	Mets	1	SD/PPS	4.4+	5.2	ND	ND	ND
10A	43	SCC	18	Mets	2	PD	1.4	4.3	**-**	**+**	**+**
11A	44	ACA	18	Mets	2	PD	1.5	4.9	**+**	**-**	**+**
12A	53	SCC	18	Mets	2	PD	1.6	11.1	**-**	**+**	**+**
13A	36	SCC	18	Mets	6	PD	5.6	10.7	**-**	**+**	**+**
14A	36	SCC	18	NED	6	PD	5.3	20.5	NA	-	-
15A	38	SCC	18	NED	10	NED	67.1+	67.1+	**-**	**+**	**+**
16A	36	SCC	18	Mets	14	SD	115.9+	115.9+	**NA**	**+**	**+**
17A	34	SCC	16	Mets	4	PD	0.4	3.9	**NA**	**+**	**+**
18A	41	SCC	16	Mets	3	PD	1.5	3.7	**NA**	**-**	**-**

*Abbreviations:* *Pt* Patient, *Path* Pathology, *#Vac* number of administered vaccines, *IR* Immune Response, *ND* Not done, *NA* Not available, *Cr-51* Chromium-51 release assay, *ELISPOT* Enzyme-Linked ImmunoSpot, *SCC* Squamous Cell Carcinoma, *ACA* Adenocarcinoma, *ASCC* Adenosquamous Carcinoma, *NED* No Evidence of Disease. *PD* Progression of Disease, *PPS* Poor Performance Status, *SD* Stable Disease, *Mets* metastatic disease, *PFS* Progression Free Survival, *OS* Overall Survival, *ms* Months.

**Table 2:** **HPV16 E7 Patient profile, clinical and immmunological outcome**

**Pt**	**Age**	**Path**	**HPV type**	**Disease extension**	***#Vac***	***Off-Study response/reason***	***PFS (ms)***	***OS (ms)***	***Cr-51***	***ELISPOT***	***IR***
1B	37	ACA	16	Mets	9	PD	10.0	25.2	**+**	**N/A**	**+**
2B	40	SSC	16	Mets	5	PD	6.3	19.9	**+**	**N/A**	**+**
3B	41	SSC	16	Pelvis	4	PD	4.6	8.5	**+**	**+**	**+**
4B	33	SSC	16	Pelvis	2	PD	2.4	13.9	-	NA	-
5B	53	SSC	16	Mets	4	PD	1.6	7.2	**-**	**+**	**+**
6B	37	SSC	16	Pelvis	1	PD	1.5	4.4	ND	ND	ND
7B	48	SSC	16	Mets	4	PD	1.7	6.3	-	-	-
8B	53	SSC	16	Mets	4	PD	3.6	42.6	-	-	-
9B	37	SSC	16	Pelvis	6	PD	5.3	7.4	**-**	**+**	**+**
10B	68	SSC	16	Pelvis	3	PD	2.5	5.5	**-**	**-**	**-**
11B	49	SSC	16	Mets	8	PD	7.7	7.7+	**+**	**+**	**+**
12B	51	SSC	16	Mets	2	PD	1.6	18.4	**-**	**-**	**-**
13B	45	SSC	16	Mets	6	SD/W	7.5+	7.5+	**-**	**+**	**+**
14B	28	SSC	16	Mets	4	PD	3.5	11.5	NA	NA	NA

*Abbreviations:* *Pt* Patient, *Path* Pathology, *#Vac* number of administered vaccines, *IR* Immune Response, *ND* Not done, *NA* Not available, *Cr-51* Chromium-51 release assay, *ELISPOT* Enzyme-Linked ImmunoSpot, *SCC* Squamous Cell Carcinoma, *ACA* Adenocarcinoma, *ASCC* Adenosquamous Carcinoma, *NED* No Evidence of Disease. *PD* Progression of Disease, *PPS* Poor Performance Status, *SD* Stable Disease, *Mets* metastatic disease, *PFS* Progression Free Survival, *OS* Overall Survival, *ms* Months.

### Peptide selection

Using computer-based prediction web sites such as the syfpeithi [(http://www.syfpeithi.de/Scripts/MHCServer.dll/EpitopePrediction.htm)] and the Bimas [(http://thr.cit.nih.gov/molbio/hla_bind/)], the HLA-A2 restricted epitopes in the E6 and E7 sequence were detected. E6 (18–26) peptide (PR-KLPQLCTEL-LYS-9-LEU) and E7 (12–20) peptide (MLDLQPETT-MET-9-THR) were the strongest A2 binders. Therefore, they were used in this trial.

### Vaccine preparation

Pre-immature Dendritic cells (PIDCs) were prepared from peripheral blood mononuclear cells (MNCs) obtained by either automated leukapheresis (Aph) or whole blood (WB) collection. The sequence of MNC collection for vaccine preparation in each patient was Aph, Aph, WB, WB, with repetition of this sequence for subsequent cycles. For Aph cycles, patients underwent leukapheresis on the Fenwal CS3000 blood cell separator using anticoagulation with ACA-A and peripheral or central venous access, with a volume processed of 3–5 liters. MNCs were purified from the leukapheresis collection by an automated ficoll-hypaque density gradient procedure, and 2 × 10^9^ cells were placed into culture for 2 days with RPMI 1640 (Cambrex, Walkersville, MD) supplemented with GM-CSF, 0.4 ng/ml, and 5% autologous heat-inactivated plasma in 162 cm^2^ flasks (Costar, Myriad, San Diego, CA), at 37°C, with 5% CO2. After 40–46 hours, cells were concentrated to 2 × 10^7^/ml, and 1 × 10^9^ cells were pulsed with the HPV16 E6(18–26) peptide (PR-KLPQLCTEL-LYS-9-LEU) or HPV16 E7 (12–20) peptide (MLDLQPETT-MET-9-THR), 10 μmolar, for 2 hours, washed, and resuspended in PlasmaLyte A with 2% autologous plasma or serum to a volume of 25–40 ml, and irradiated with 25 Gy from a Cesium source. For WB cycles, 100–150 ml of peripheral venous blood was drawn into heparin sulfate (10 units/ml blood). MNCs were separated on a ficoll hypaque gradient, washed and resuspended. 70 × 10^6^ cells were cultured at a concentration of 4–5 × 10^6^/ml in RPMI 1640 (Cambrex) with 5% heat-inactivated autologous serum or allogeneic AB human serum, supplemented with 0.4 ng/ml of GM-CSF at 37°C with 5% CO2. The HPV16 E6 peptide, 10 μmolar, was added at initiation of culture, and was therefore present for the entire 2 day culture duration. After 40–46 hours, the peptide-pulsed cells were harvested, washed, and resuspended in PlasmaLyte A with 2% autologous serum or allogeneic AB serum to a volume of 6 ml, and irradiated with 25 Gy. For Aph cycles, infusion of up to 1 × 10^9^ peptide-pulsed PIDCs was allowed, with a cell concentration of 20 × 10^6^/ ml, whereas the WB cycles allowed a maximum of 70 × 10^6^ cells, with a cell concentration of 10 × 10^6^/ml.

### Vaccine administration

Prior to vaccine administration, peptide hypersensitivity testing was done. Briefly, 1 μg of peptide dissolved in 0.1 ml of preservative-free normal saline was injected intradermally in the outpatient clinic, the patient was observed for 1 hour. The peptide-pulsed PIDCs were infused intravenously (IV) at a rate of 5 ml over 1–2 min through a sterile 110 micron filter needle. Vaccination was repeated at week 3, then every 4 weeks for a total of 14 vaccinations or until disease progression. Patients were observed for another hour after vaccination.

### Clinical monitoring

Patients were evaluated for toxicity and tumor response during treatment and up to two years after the last vaccination. Tumor response was assessed by physical exam and by the appropriate imaging technique (CT-scan) according to RECIST criteria at baseline, then every 2 vaccinations during therapy and every 3 months during follow-up. Patients were taken off protocol because of either deterioration in the performance status, disease progression or withdrawal from the study. Disease progression was defined per the modified WHO criteria of progression as the appearance of new lesions and/or a 25% increase of measurable lesions as evident by CT-scan. Once patients had progressed, follow-up was not required except to document late toxicities and death. Adverse events and toxicities were defined and graded according to the NCI Common Toxicity Criteria.

### Immunological testing

To assess the immune response, lymphocytes were isolated from PBMC. Immunologic testing was performed using Chromium-51 release assay (51Cr release assay) against cervical cancer cell lines as described below and Enzyme-Linked ImmunoSpot (ELISPOT). The post-vaccination samples were tested at the date of the next administered vaccines prior to each vaccination and compared to pre-vaccination samples. The pre-vaccination and post-vaccination samples were frozen and assayed at the same time. The immune response was considered positive for patients who demonstrated a positive immune response by either ELISPOT, 51Cr release assay or both.

#### Cell lines

Cervical cancer cell lines used in this trial were either from the ATCC (Mannassas, VA) including the ME180, Caski and MS751 or established at the NCI (Bethesda, MD) like the Cav cervical cancer cell line. All cells were tested in our lab for HLA and HPV typing. Caski, MS751 and Cav are all HLA-A2 positive cell line while ME180 is an A2 negative cell line. Caski harbors the HPV16 genome while MS751 and the Cav harbor the HPV18 genome (low and high number of copies respectively). All cells grew in RPMI supplemented with 10% FCS, Penicillin, L-Glutamine and Sodium Pyruvate.

#### Chromium-51 release assay (51Cr release assay)

The cervical cancer cell lines mentioned above were used as targets in the cytotoxic T lymphocyte (CTL) assay. The cytolytic activity was measured by using the 4-hour 51Cr release assay. Lymphocytes were prepared from PBMC. These cells were selected and expanded in vitro in the presence of 1–20 μM of E6 peptide with 5 IU/ml of IL-2 (Chiron, Emeryville, CA) added on day 3 for 7 days. This 7 day cycle was repeated one more time, after which the cells were harvested and tested for specific cytolytic activity against E6 or E7 peptide pulsed autologous APC’s (EBV transformed B cells) or tumor cell lines harboring the HPV genome. Target cells were washed with RPMI medium and labeled with 200 μCi ^51^Cr sodium dichromate in the presence or absence of 10 μM of E6 or E7 peptide for 2 hours, after which target cells were washed and plated in 96 well round bottomed plates. Effectors were added to labeled targets at the desired effector to target ratio. The plates were centrifuged at 500 rpm for 5 min., and incubated at 37°C for 4 hours. Supernatants were harvested using a Skatron supernatant harvesting device, and the samples counted on a gamma counter. The percent specific lysis was determined, on triplicate samples, by the following formula:

$$ \mathrm{Percent}\ \mathrm{specific}\ \mathrm{lysis} = \mathrm{experimental}\ \mathrm{release}\ \hbox{-}\ \mathrm{spontaneous}\ \mathrm{release} \times 100 $$

#### Maximum release - spontaneous release

A result was considered positive with a two fold increase or more in lytic units pre-immunization. If there was no detectable pre-immunization specific lysis, a post immunization lysis of greater than 10% above the non-peptide was considered positive, with an Effector:Target (E:T) ratio specified at 50:1. If an E:T ratio of 50:1 was not tested, the next highest ratio was used. In some patients we established CTL and tumor cell lines for future use in studies of antigen specificity.

#### Enzyme-linked ImmunoSpot (ELISPOT) assay

ELISPOT assays were performed at the Laboratory of Cell-Mediated Immunity, SAIC-Frederick (CLIA-certified lab). Two frozen normal donor controls with known responsive values were run with each assay to assure quality control of the assay results. For all assays, at least one of the two controls was within 2 standard deviations of the laboratory-generated means for CMV and CEF. All assays were performed on 7–8 day in vitro stimulated PBMCs (100 K/well) as the effectors and peptide-pulsed autologous PBMCs (100 K/well) as the antigen presenting cells (APCs). When possible, PBMCs from the earliest time point were used as the APCs. However, if this was not possible, the pulsed PBMCs were assayed alone to make sure they were not producing any spots. Briefly, the day before assay setup, 96-well polyvinylidene fluoride (PVDF) membrane, HTS opaque plates (Millipore, Billerica, Massachusetts, MSIPS4W10) were coated overnight with a 1:100 dilution of anti-human IFN-γ capture antibody (1 mg/mL, Mabtech Inc., Mariemont, OH, Cat# 3420-3-1000) in Dulbecco’s phosphate buffered saline (DPBS) at room temperature. Antibody-coated plates were washed four times in DPBS the next day and blocked with 5% human AB ELISPOT medium at 37°C for approximately 2 hours. 1 × 105in vitro stimulated PBMCs and 1 × 105 autologous, peptide-pulsed PBMCs were plated per well. The plates were incubated for 18–20 hours at 37°C. The next day, the plates were manually washed six times with 0.05% Tween 20 in DPBS, followed by a 2-hour incubation at room temperature with a 1:2000 dilution of the biotinylated secondary antibody, anti-human IFN-γ (1 mg/mL Mabtech Inc., Mariemont, OH, Cat# 3420-6-1000) in DPBS/1% bovine serum albumin/0.05% Tween. After incubation and four washes in DPBS to remove excess antibody, a 1:3000 dilution of streptavidin alkaline phosphatase (Mabtech, Mariemont, OH, Cat# 3310–10) in DPBS/1% bovine serum albumin, was added to each well for 1 hour at room temperature followed by 4 manual washes in DPBS. Finally, the BCIP/NPT substrate, 100 μl/well, (KPL, Gaithersburg, Maryland, Cat# 50-81-08) was added for 7–10 minutes, resulting in the development of spots. The reaction was stopped by washing three times in distilled water. Plates were dried overnight and the spots were visualized and counted using the ImmunoSpot Imaging Analyzer system (Cellular Technology Ltd., Cleveland, OH). ELISPOT results were expressed as the “number of spots per 10^6^ responder cells” after subtracting background spots obtained in wells of effectors with non-pulsed PBMCs. For each subject, PBMCs obtained before and after vaccination were analyzed in the same assay to avoid inter-assay variability. A post-immunization fold increase >2 compared to pre-immunization was considered a positive response.

### Statistical analysis

This study was carried out as a pilot trial to gain experience administering vaccines with the E6 and E7 peptide. The primary objectives of this study was to determine whether vaccinating advanced cervical cancer patients with Pre-Immature Dendritic Cells (PIDC) pulsed with HPV16 E6 or E7 peptide is safe and capable of eliciting specific immune response against the relevant administered peptide. It was intentionally small in order to have adequate numbers for evaluation of feasibility and immune response, but limited information on clinical efficacy. All results presented are intended to be hypothesis generating in view of the intention of the trial and the limited number of subjects treated.

As secondary outcomes from the trial, overall survival survival (OS) and progression free survival (PFS) were computed from the date the consent was signed until the date the patient died, progressed or was last followed as appropriate. The Kaplan-Meier method was used to estimate the probability of survival or progression free survival as a function of time.

## Results

### Patient profile

Thirty-two patients with advanced cervical cancer were enrolled on this trial. Eighteen patients received the HPV1A6 E6 (18–26) peptide (arm A) and 14 patients received the HPV16 E7 (12–20) peptide (arm B). The patients’ characteristics are summarized in Table 1 (Arm A) and Table 2 (Arm B). Age ranged from 28 to 68 years with a mean of 40.8 in arm A and 44.2 in arm B. Twenty six patients had squamous cell carcinoma (13 in each arm), 4 patients had adenocarcinoma (3 in arm A and 1 in arm B), and 2 patients in arm A had adeno-squamous carcinoma. In arm A, four patients’ tumors harbored HPV16 and fourteen patients’ tumors harbored HPV18. Whereas in arm B, all patients’ tumors harbored the HPV16 subtype. Patients in both arms were heavily pretreated; Twenty two patients underwent at least one surgery with a course of radiation. Two patients received at least one surgery with a course of chemotherapy. The remaining eight patients received only radiation and chemotherapy. Patients had measurable disease on enrollment except patients 14A and 15A who had no evidence of disease. Patient 14A had stage IB on diagnosis which was treated with surgery followed by adjuvant radiation therapy. Subsequently, further treatment with radiation therapy and chemotherapy was given for recurrent disease in the para-aortic lymph nodes. Patient 15A was diagnosed with stage IIB during laparotomy, and treated with adjuvant radiation therapy. However, this patient developed pulmonary metastasis and was treated with radiation and chemotherapy followed by thoracotomy.

### Immunological data

#### Phenotype of the pre-immature dendritic cells (PIDCs)

**Figure 1:**
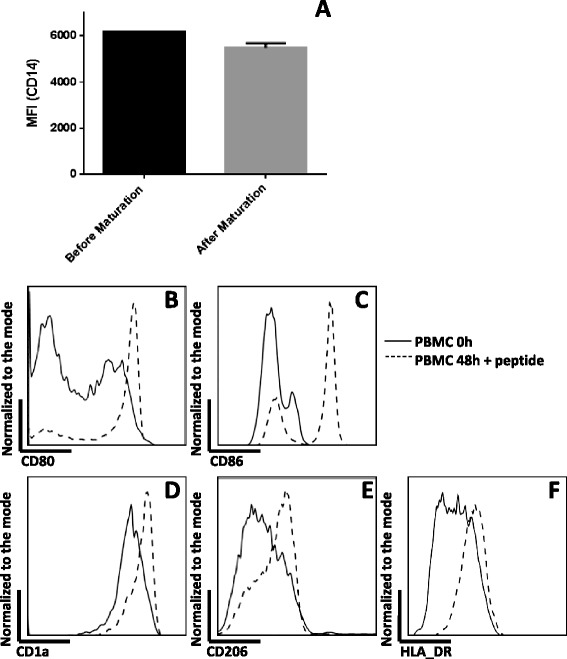
**FACS analysis showing the phenotype of PIDCs used in the vaccine preparation. A)** After a two day incubation in the presence of GM-CSF, and pulsating with HPV E7 peptide for two hours, CD14 positive cells persistently expressed CD14 at a slightly lower level. **B)** CD14 positive cells expressed a higher level of CD80 following incubation with GM-CSF and HPV E7 peptide. **C)** CD14 positive cells expressed a higher level of CD86 following incubation with GM-CSF and HPV E7 peptide. **D)** CD14 positive cells expressed a higher level of CD1a following incubation with GM-CSF and HPV E7 peptide. **E)** CD14 positive cells expressed a higher level of the Mannose receptor (CD206) following incubation with GM-CSF and HPV E7 peptide. **F)** HLA-DR was up-regulated in CD14 positive cells following incubation with GM-CSF and HPV E7 peptide.

FACS analysis was done on the PIDCs before vaccine administration to document their phenotype. The data was further confirmed using PBMCs from healthy donors. Prior to the GM-CSF incubation; the PIDCs were all monocytes expressing CD14 and they did not express any of the antigen presenting cell surface markers. After two days in culture in the presence of GM-CSF, and pulsing for two hours with the HPV16 E6 or E7 peptide, in spite of the persistent expression of CD14 at slightly lower levels (Figure 1A), CD14 positive cells (both from PIDCs and PBMCs) were found to express antigen presenting cell associated surface markers (CD80, CD86, CD1a and Mannose receptor (CD206)) (Figure 1B-E). Furthermore, there was up regulation in HLA-DR (Figure 1F).

#### The cross reactivity between HPV16 E6 and HPV18 E6

**Figure 2:**
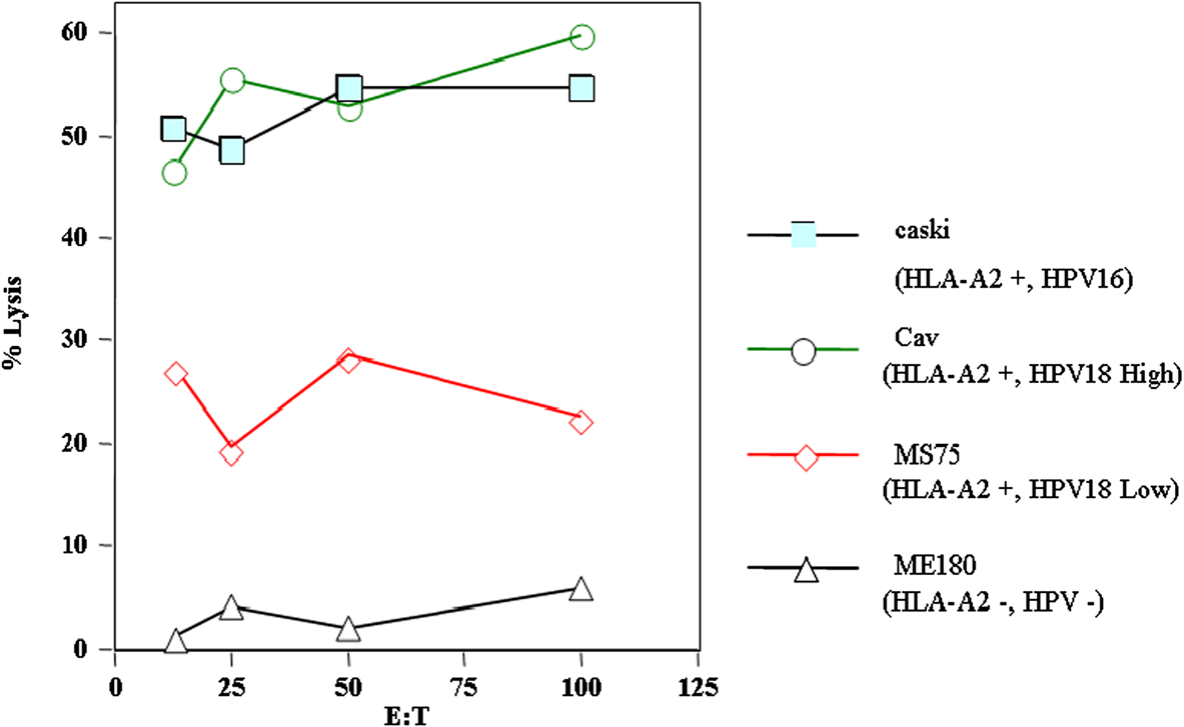
**The cytolytic activity of patient 6A’ PBMCs post one vaccination.** Cervical cancer cell lines were used as target cells. Caski (HPV16 positive, HLA-A2 cell line) closed squares; CAV (HPV 18 high and HLA-A2 cell line), open circles; MS751 (an HPV 18 low, HLA-A2 cell line), open diamonds; ME180 (HPV negative, HLA-A2 negative cervical cancer cell line), open triangles. Highest percentage of specific lysis was seen with the Caski and Cav cell lines, followed by the MS751 and only background level lysis was seen when the ME180 cell line was used as a target.

To test whether E6 protein is processed and presented in the proper context of MHC class I and to test the ability of T-cells generated by HPV16 E6 to cross react with an internally processed HPV18 E6, we tested the ability of post vaccination PBMCs to lyse established HLA-A2 positive cervical cancer cell lines harboring either the HPV16 or HPV18 genome. We found that these primed T-cells were able to lyse Caski cells (HLA-A2 and HPV16 positive), Cav cell line (HLA-A2 with a high number of copies of HPV18) and, to a lesser extent, the MS751 cell line (HLA-A2 harboring lower number of copies of HPV18) but not the HLA-A2 and HPV negative cervical cancer cells (ME180). We were able to see specific lysis in those cell lines and the lysis was higher in cell lines that carry higher number of copies of HPV (Figure 2). Similarly, the effector cells generated through the E7 vaccine were tested against tumor cells either expressing the HPV16 E7 genome or HPV16 E7 negative cell line. We were able to detect lysis of the E7 positive cell lines and not the E7 negative ones. Furthermore, effector cells were able to lyse only the HLA-A2 positive cell lines and not the HLA-A2 negative ones (data not shown).

#### Immune response

Immunological responses were evaluated in 28 out of 32 treated patients (Tables 1 and 2). Three patients were excluded from the analysis since they received only one vaccine due to disease progression (2A, 9A, 6B), and one patient (14B) was not tested for an immune response due to lack of sufficient cell numbers to reproduce the results. Seventeen out of the 28 (61%) evaluated patients had positive immune responses by either ELISPOT, 51Cr release assay, or both [10/16 (63%) in arm A, and 7/12 (58%) in arm B]. The immune response data for both ELISPOT and 51Cr release assay are presented in Additional file 1: Figures S1 and Additional file 2: Figure S2.

### Clinical response

**Figure 3:**
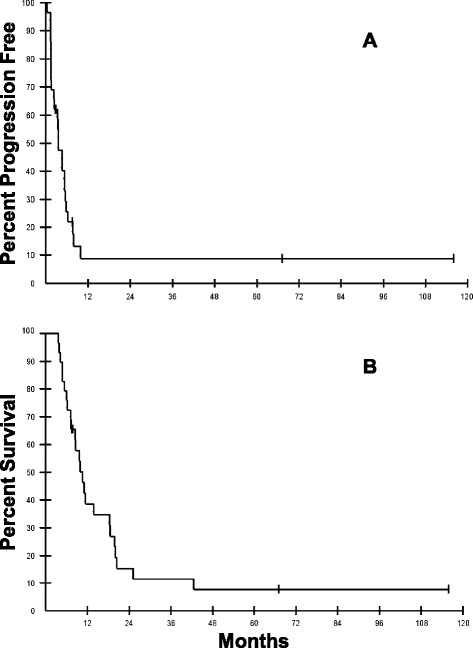
**Progression free survival and overall survival for the full cohort.** Progression free survival **(A)** and overall survival **(B)** for the full cohort. PFS was calculated as time from the date of consent until evidence of disease progression or last follow-up. OS was calculated as time from consent date until death or last follow-up.

Clinical response was evaluated in 29 out of the 32 treated patients (Tables 1 and 2). Three patients (2A, 9A and 6B) were excluded from the analysis since they received only one vaccine due to poor performance status. Out of the 29 evaluable patients the clinical outcome was as follows: Twenty five patients had progression of disease during the course of treatment (13/16 on arm A and 12/13 patients on arm B); one patient (5A) was taken off the study due to poor performance status after receiving 2 vaccine doses; one patient (#13B) decided to withdraw from the study and had a stable disease after receiving 6 vaccine doses; and the remaining 2 patients (#15A and 16A) completed the treatment without disease progression. Patient 15A was enrolled on the study with NED after being treated with 2 surgeries, 2 courses of radiation therapy and one chemotherapy regimen. This patient received a total of 10 vaccine doses and continued to have no evidence of disease as of the last follow up (67.1 months). Patient 16A was enrolled on the study with stage IV disease after being treated with radiation therapy and 2 courses of chemotherapy. This patient completed a total of 14 vaccine doses and continued to have a stable disease, as of the last available follow up (115.9 months). There were no RECIST responses in any patient. The median overall survival (OS) and progression free survival (PFS) for the full cohort was 10.0 and 3.5 months, respectively (Figure 3). Arm A had a median OS and PFS of 9.7 and 3.6 months, respectively, while patients on arm B had a median OS and PFS of 11.4 and 3.5 months, respectively. Eighteen patients received fewer than five vaccine doses and had a median OS and PFS of 7.2 and 1.7 months, respectively. On the other hand, 11 patients who were able to receive five or more vaccine doses had a median OS and PFS of 20.1 and 6.9 months, respectively. Patients who had an immune response had a median OS and PFS of 8.7and 5.3 months, respectively, while the non-immune responders had a median OS and PFS of 6.3 and 2.8 months, respectively.

### Safety and toxicity

The vaccine was well tolerated. No allergic reaction to the vaccine was reported in either arm. The majority of toxicities were grade I or II, with fatigue being the most common occurring in 12/18 (67%) patients in arm A and 6/14 (43%) of patients in arm B. Grade III toxicities were reported in both arms, majority of which were attributed to the disease process. These events included: anemia, nausea, vomiting, shortness of breath, chest pain, pyelonephritis, and thrombocytopenia. No grade IV toxicity was reported on either arm (Table 3).

**Table 3:** **Toxicities**

**Toxicity grade 3**	**# of patients**
Anemia	1 (13A)
Nausea	1 (17A)
Vomiting	1 (17A)
SOB	1 (18A)
Chest pain	1 (18A)
Pyelonephritis	1 (9A)
Thrombocytopenia	1 (14B)

*Abbreviations:* *SOB* shortness of breath.

## Discussion

In this pilot study Pre-Immature Dentritic Cells (PIDC) pulsed with HPV16 E6 (18–26) or HPV16 E7 (12–20) peptides were found to be capable of generating specific immune response as evident by the 51Cr release CTL or ELISPOT assay. The majority of patients on both arms had a measurable increase in the number of IFN-γ secreting cells post vaccination. In the cytolytic assay, we were able to show that PBMCs of patients who received the vaccine were able to recognize and lyse target cells pulsed with the E6 or E7 peptide. In addition, we showed that the HPV16 E6 is homologous to HPV18 E6 and cross-react. We were able to demonstrate that the immune response induced by HPV16 E6 can specifically lyse cervical cancer cell lines harboring either HPV16 (caski cell line) or HPV18 (Cav and MS751) *in vitro*. These findings were also evident *in ex vivo* since patients in the E6 arm developed an immune response against HPV16 E6 peptide whether they expressed HPV16 or 18 on their tumors. Accordingly, since those two peptides are able to cross react either of them can be used for vaccination of both groups of patients with HPV16 and HPV 18 expressing tumors.

The antigen presenting cells used in this trial were autologous PBMCs cultured only for 48 hours in the presence of GM-CSF, and hence we called them “Pre-Immature Dendritic Cells (PIDCs)”. In our trial, 61% of patients developed immune response against the relevant peptide indicating that the PIDCs were able to function normally and proved to be potent APCs, even though they don’t express all surface markers of DCs. These PIDCs possess a great advantage over regular DCs as they can be generated in a shorter time, can give a higher cell yield, and are not dependent on the cytokines in the culture such as IL-4.

Two previously conducted clinical trials demonstrated the immunogenicity of the recombinant E7 protein pulsed on mature DCs in advanced cervical cancer patients [21,22]. Ferrara et al. treated patients with recombinant HPV16 E7 or HPV18 E7 protein pulsed on mature DCs and administered subcutaneously and demonstrated the generation of immune response in 3 of the 11 treated patients (27%) [21]. Santin et al. treated patients with recombinant HPV16 E7 or HPV18 E7 protein pulsed on mature DCs and administered subcutaneously in combination with low dose IL-2 and showed positive immune responses in all tested patients (4/4, 100%) [22]. A live recombinant vaccinia virus expressing the E6 and E7 proteins of HPV16 and 18 was also investigated as a therapeutic vaccine in the advanced cervical cancer setting [23]. Only 1/3 (33%) of evaluated patients had a positive CTL response following re-stimulation with E6 and E7 peptides. More recently, Melief et al. investigated a mix of long peptides from the HPV-16 E6 and E7 in incomplete Freund’s adjuvant in 20 patients with HPV16 positive, high-grade vulvar intraepithelial neoplasia. Fifteen of 19 patients (79%) had clinical responses and all patients had vaccine-induced T-cell responses [24]. Indeed, it would be difficult to compare our findings to others given the different methods of vaccination and the variety of immune assays used to detect the immune response.

Although this trial was not powered to test for clinical efficacy, it revealed some interesting clinical findings. For the full cohort, the median overall survival (OS) and progression free survival (PFS) was 10.0 and 3.5 months, respectively which is encouraging given the poor expected survival in this population of patients when treated with standard chemotherapy (median OS of 4.9 months) [3]. Moreover, it is noticeable that patients who received five or more vaccine doses had a longer median OS and PFS of 20.1 and 6.9 months, respectively. Because this is not a randomized study, it would be difficult to determine whether the longer survival in this group of patients is due the higher number of vaccine doses they received or to their tumors’ biology which allowed them to survive longer and therefore to receive more vaccines. Furthermore, the disconnection between the immune and the clinical response could be related to the expansion of inhibitory immune cells such as T-regulatory cells (T-regs) and myeloid-derived suppressor cells (MDSCs) or the activation of other immunosuppressive pathways such as the PD-L1/PD-1 pathway. However, because of the small cohorts tested on this trial we cannot conclude a relationship between OS or PFS and immune response.

In summary, this pilot study represents a proof of concept for using pre-immature dendritic cells (PIDCs) for peptide delivery in cancer vaccine development. This method should be further investigated in future clinical trials. In addition, future directions should focus on investigating this method of peptide vaccination in a population of patients with early stage of disease in order to allow time for the generated immune response to be translated to a clinical effect. Combining this method of vaccination with other modalities such as chemotherapy, radiation therapy or immune modulators should be also considered in the future.

## Conclusion

In summary, our trial confirmed the feasibility and safety of using HPV16 E6 and E7 peptide vaccine as a personalized treatment for patients with advanced cervical cancer. These peptide vaccines were capable of generating a specific immune response against the relevant peptide. Further studies are needed to test these peptide vaccines in early disease setting and in combination with other modalities such as immune modulators.

## Additional files

Additional file 1: Figure S1. Immune responses in patients on arm A (HPV16 E6). Immune responses of patients on arm A to HPV16E6 peptide in red compared with no peptide in blue. Immune responses were measured by ELISPOT (Panel A) or 51Cr release assay (Panel B). Abbreviations: Pre-vaccine, Pre-vaccination sample; Post-V, Post-vaccination sample marked by the vaccine number; f/u, Follow up sample marked in months (ms) from the last post vaccine sample. Additional file 2: Figure S2. Immune responses in patients on arm B (HPV16 E7). Immune responses of patients on arm B to HPV16E7 peptide in red compared with no peptide in blue. Immune responses were measured by ELISPOT (Panel A) or 51Cr release assay (Panel B). Abbreviations: Pre-vaccine, Pre-vaccination sample; Post-V, Post-vaccination sample marked by the vaccine number; f/u, Follow up sample marked in months (ms) from the last post vaccine sample.

## Abbreviations

PIDC: Pre-immature dendritic cells
HPV: Human papilloma virus
PBMCs: Peripheral blood mononuclear cell
GM- CSF: Granulocyte macrophage colony-stimulating factor
51Cr release: Chromium-51 release assay
ELISPOT: Enzyme-linked ImmunoSpot
OS: Overall survival
PFS: Progression free survival
RECIST: Response evaluation criteria
DCs: Dendritic cells
IL: Interleukin
ECOG: Eastern cooperative oncology group
NCI: National cancer institute
NNMC: National naval medical center
IRBs: Institutional review boards
MNCs: Peripheral blood mononuclear cells
Aph: Automated leukapheresis
WB: Whole blood
IV: Intravenously (IV)
CTL: Cytotoxic T Lymphocyte
TLN: Tumor-draining lymph nodes
APC’s: Antigen presenting cells
IFN-gamma: Interferon-gamma
