# Supplementary figures and images for: Pre-immature dendritic cells (PIDC) pulsed with HPV16 E6 or E7 peptide are capable of eliciting specific immune response in patients with advanced cervical cancer
Source: J Transl Med. 2014 Dec 16;12:353. doi: 10.1186/s12967-014-0353-4 (PMC4269078; doi:10.1186/s12967-014-0353-4)

## Slide 1
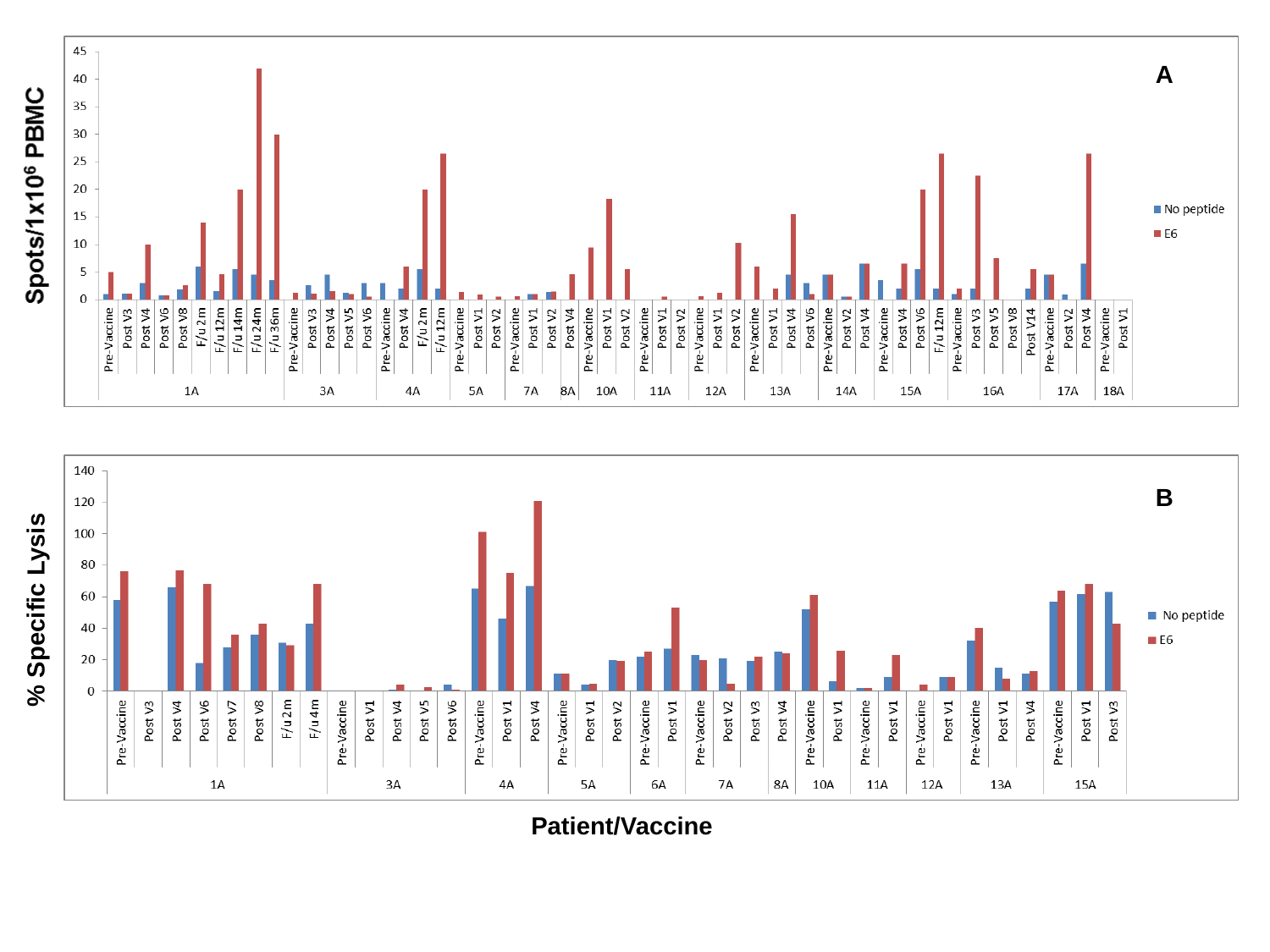

A
B
% Specific Lysis
Patient/Vaccine

Supplement: Additional file 1: Figure S1. — Immune responses in patients on arm A (HPV16 E6). Immune responses of patients on arm A to HPV16E6 peptide in red compared with no peptide in blue. Immune responses were measured by ELISPOT (Panel A) or 51Cr release assay (Panel B). Abbreviations: Pre-vaccine, Pre-vaccination sample; Post-V, Post-vaccination sample marked by the vaccine number; f/u, Follow up sample marked in months (ms) from the last post vaccine sample. [file 12967_2014_353_MOESM1_ESM.pptx]

## Slide 1
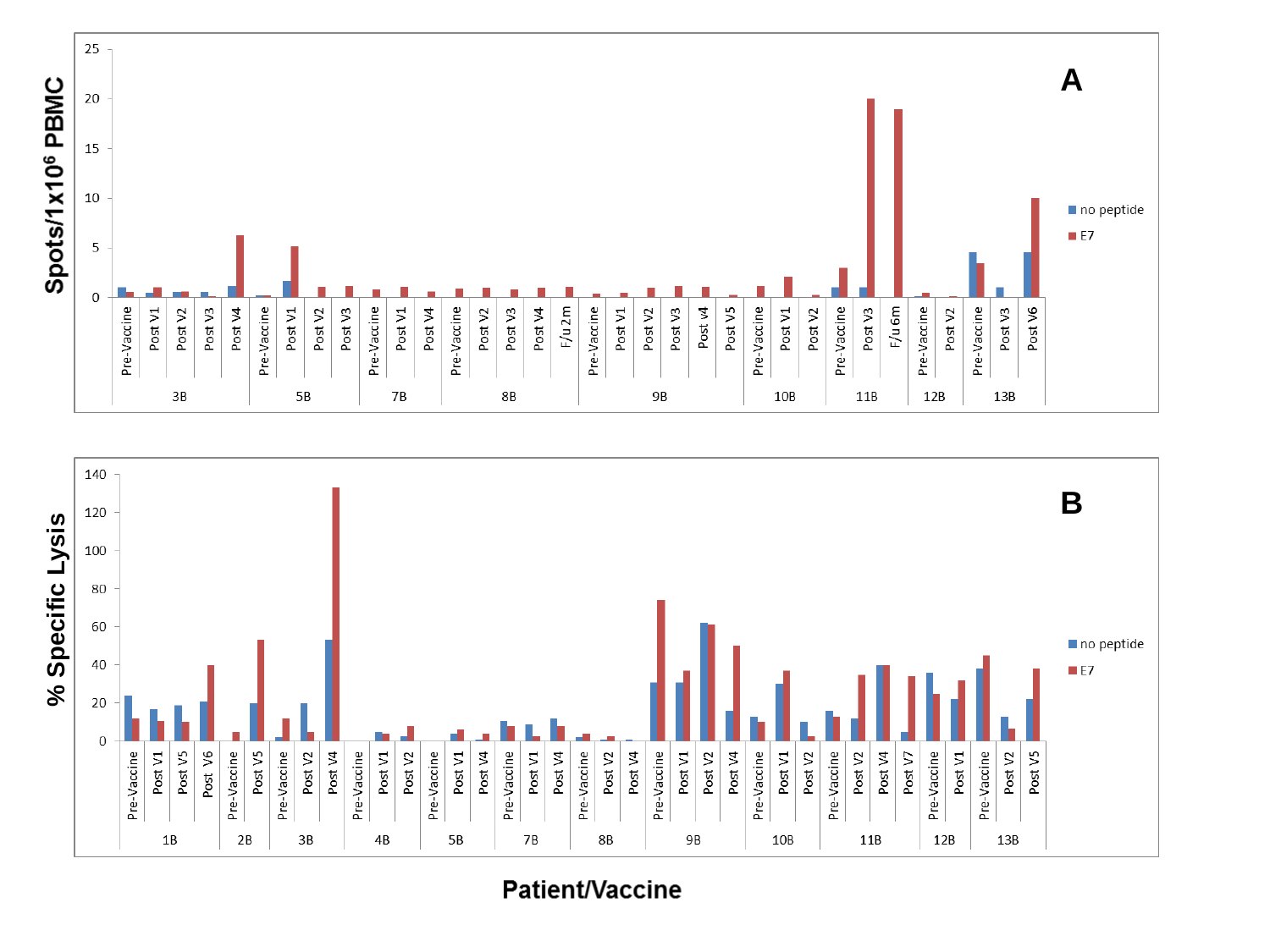

A
B
% Specific Lysis

Supplement: Additional file 2: Figure S2. — Immune responses in patients on arm B (HPV16 E7). Immune responses of patients on arm B to HPV16E7 peptide in red compared with no peptide in blue. Immune responses were measured by ELISPOT (Panel A) or 51Cr release assay (Panel B). Abbreviations: Pre-vaccine, Pre-vaccination sample; Post-V, Post-vaccination sample marked by the vaccine number; f/u, Follow up sample marked in months (ms) from the last post vaccine sample. [file 12967_2014_353_MOESM2_ESM.pptx]
